# Amplified Fluorescence by ZnO Nanoparticles vs. Quantum Dots for Bovine Mastitis Acute Phase Response Evaluation in Milk

**DOI:** 10.3390/nano10030549

**Published:** 2020-03-18

**Authors:** Narsingh R. Nirala, Giorgi Shtenberg

**Affiliations:** Institute of Agricultural Engineering, ARO, the Volcani Center, Bet Dagan 50250, Israel; niralamn07@gmail.com

**Keywords:** mastitis biomarker, *N*-acetyl-β-d-glucosaminidase, nanoparticles, quantum dots, signal enhancement, zinc oxide

## Abstract

Bovine mastitis (BM) is a prominent inflammatory disease affecting the dairy industry worldwide, originated by pathogenic agent invasion onto the mammary gland. The early detection of new BM cases is of high importance for infection control within the herd. During inflammation, various biomarkers are released into the blood circulation, which are consequently found in milk. Herein, the lysosomal activity of *N*-acetyl-β-d-glucosaminidase (NAGase), a predominant BM indicator, was utilized for highly sensitive clinical state differentiation. The latter is achieved by the precise addition of tetraethyl orthosilicate-coated zinc oxide nanostructures (quantum dots or nanoparticles, individually) onto a conventional assay. Enhanced fluorescence due to the nanomaterial accumulative near-field effect is achieved within real milk samples, contaminated with *Streptococcus dysgalactiae*, favoring quantum dots over nanoparticles (>7-fold and 3-fold, respectively), thus revealing significant differentiation between various somatic cell counts. The main advantage of the presented sensing concept, besides its clinically relevant concentrations, is the early bio-diagnostic detection of mastitis (subclinical BM) by using a simple and cost-effective experimental setup. Moreover, the assay can be adapted for BM recovery prognosis evaluation, and thus impact on udder health status, producing an alternative means for conventional diagnosis practices.

## 1. Introduction

Bovine mastitis (BM) is a prominent inflammatory disease affecting the dairy industry worldwide, originated by pathogenic agent invasion onto the mammary gland [1,2,3]. Decreased milk production yield, unsatisfying product quality and increased treatment costs are all proportionally correlated to the severity of the occurring BM [4,5]. The disease is classified as chronic, clinical and subclinical based on the induced pathogenesis [2,6,7]. The latter (>90% of BM cases) is difficult to detect due to the absence of any visible indications, as in the case of clinical BM (visual milk abnormalities, tissue swelling and redness, systemic illness, etc.) [1,2]. Conventional techniques measure somatic cell counts (SCC), identify causative pathogenic bacteria by selective cell culture diagnosis and assess enzymatic activity [3,5,8,9]. Lactate dehydrogenase and *N*-acetyl-β-d-glucosaminidase (NAGase) are predominant indicators of BM, which correlate with the SCC levels found in milk [3]. These enzymes are released upon damage to or lysis of epithelial cells of the mammary gland, therefore representing augmented health status [1,6]. NAGase peripheral distribution in plasma increases (up to an order of magnitude) upon inflammation or infection with respect to healthy conditions [1,3,10,11]. NAGase lysosomal activity is used to differentiate healthy quarters from all other inflamed statuses based on a fluorescence (FL) methodology developed by Kitchen et al. [2,6,12]. Briefly, the method includes the specific lysosomal breakdown of 4-methylumbelliferyl-*N*-acetyl-β-d-glucosaminide (4-MUAG) substrate to fluorogenic products of 4-methylumbelliferone (4-MU) in alkaline conditions [6,12]. Despite the known advantages of the procedure, e.g., being simple, rapid and affordable, it suffers from low quantum efficiency and low sensitivity in differentiating subclinical cases [13]. Our previous report has attempted to overcome these limitations by employing silica-coated zinc oxide (ZnO) quantum dots (QDs) for enhanced FL of the obtained 4-MU product by the conventional NAGase assay [13]. Intensified emission values were achieved in correlation to SCC, pathogen type (coagulase-negative *staphylococci* and *Streptococcus dysgalactiae* (*Strep. dysgalactiae*)) and the severity of the occurring BM without hindering the optical performance. 

ZnO nanostructures (QDs, nanoparticles—NPs, thin-films, nanorods, nanoflowers, etc.) are widely used multifunctional semiconducting materials for complex biosensing applications utilizing their unique physical and chemical properties [14,15,16,17,18,19,20,21]. These nanostructures are optically stable at room temperature, presenting a high exciton energy (60 meV) and wide band-gap (3.36 eV) [18]. Moreover, ZnO nanomaterials absorb UV radiation and thus act as potent signal enhancers for FL-based sensors [15]. The mechanisms behind the FL enhancement are due to the evanescent electric field at the surface of the nanomaterial that increases the excitation energy of the fluorescent molecule [19]. ZnO is a low-cost material synthesized by various techniques that control size, morphology and functionality (capping agents used to modify its surface include: silanes, polyvinylpyrrolidone, aliphatic thiols and amines) [13,22]. Several reports depict the practical usage of ZnO for FL enhancement across the visible-light spectrum. Tereshchenko et al. have shown a highly sensitive optical biosensor based on ZnO thin-films for monitoring *Grapevine virus* type-A, which infects healthy plants [23]. The specific immunorecognition of the target antigen presented correlative luminescence intensity changes around the 425 nm band. The optical response depicted a wide dynamic range of 1 pg to 10 ng mL^−1^ with high selectivity and applicability for on-site detection. A similar concept using FITC-modified antibodies for cancer biomarkers’ detection has shown multiplex bio-detection capabilities [24]. Polyethylenimine-modified ZnO nanowires impregnated by microfluidics channels enhanced the inherent FL signal for human α-fetoprotein and carcinoembryonic antigen detection over 6–7 orders of magnitude, while presenting detection limits of 1 pg mL^−1^ and 100 fg mL^−1^, respectively. Akhtar et al. have modified ZnO nanoflowers grown over silver thin-films with Thioflavin T for the sensitive targeting of amyloids as an early diagnosis methodology for different neurodegenerative disorders [25]. Enhanced FL, by 9.8-fold, was obtained upon insulin amyloid binding to the nanobiosensor, reinforcing the excitation of fluorophore molecules in close proximity with ZnO. 

Herein, a comparative study evaluated the lysosomal activity of NAGase by FL signal amplification in the presence of silica-coated ZnO-QDs and ZnO-NPs. The synthesized nanomaterials were optically and chemically characterized for optimal FL signal amplification. Different milk qualities at three SCC levels, contaminated with *Strep. dysgalactiae*, influence on the secreted NAGase content, were evaluated with respect to healthy udder (with and without ZnO nanomaterials). Overall, the optical performances based on intensified FL signals offered the means to deduce the severity of the occurring BM and the overall udder health with respect to a conventional assay protocol with improved sensitivity. 

## 2. Materials and Methods 

### 2.1. Materials

Methanol, potassium hydroxide, sodium citrate dihydrate, tetraethyl orthosilicate (TEOS, 98%) and zinc nitrate hexahydrate (98%) were purchase from Merck. Ammonium hydroxide water (28%), ethanol, 4-MUAG, 4-MU, ZnO nanopowder (˂100 nm particle size) and analytical grade buffers were supplied by Sigma-Aldrich. Milli-Q water (18 MΩ cm) was used for the denoted experiments. 

### 2.2. Fabrication of TEOS Modified ZnO-QDs

ZnO-QDs were prepared by a modified protocol previously reported by Parta et al. [18]. Briefly, zinc nitrate hexahydrate (0.1 M) and potassium hydroxide (0.1 M), both methanolic solutions, were mixed for 1 h while maintaining alkaline conditions. The resulting ZnO-QDs were modified with 250 µL TEOS for optimized molecular coverage, resulting in ZnO-QDs-SiO_2_ [13]. The colloidal solution was centrifuged (10,000 rpm, 10 min) and washed with methanol three times. The final product was aqueously diluted to 6.5 mg mL^−1^ before prior use. 

### 2.3. Fabrication of TEOS Modified ZnO-NPs

ZnO-NPs were silica-coated according to a previous study by El-Nahhal et al. [16]. Briefly, 25 mg of ZnO nanopowder was dispersed in a mixture of ammonium hydroxide, ethanol and milli-Q water (0.15, 10, 5 mL, respectively), followed by 30 min sonication. Next, the dispersion was mixed with TEOS (0–100 µL for optimized molecular coverage) for 24 h at room temperature, resulting in ZnO-NPs-SiO_2_. The resulting product was centrifuged (4000 rpm, 5 min) and washed with ethanol three times. The final product was aqueously diluted to 6.5 mg mL^−1^ before prior use. 

### 2.4. Milk Sampling

Milk samples were obtained by authorized personnel from specific quarters of Holstein cows (Volcani Center research herd) and classified as healthy, subclinical and clinical BM, as previously described [10]. The Institutional Animal Care Committee of ARO approved animal experiments (838/119IL).

### 2.5. Quantification of NAGase Activity 

The quantification of NAGase activity was as previously described by Nirala et al. [13]. Milk samples (30 µL) were mixed with 40 µL of substrate (2.25 mM 4-MUAG) or 40 µL of the product (0 to 100 µM of 4-MU) in acidic conditions (citrate buffer, pH 4.6). The reaction solution was mixed on an automatic shaker for 1 min with 100 µL of optimized ZnO nanomaterial concentration (0–6.5 mg mL^−1^ for ZnO-QDs-SiO_2_ and ZnO-NPs-SiO_2_). Finally, end-point FL studies were carried using 365 nm excitation and measuring the FL after 12 min of reaction. 

### 2.6. Instrumentation

UV-VIS absorption spectra and FL studies were analyzed with Varioskan™ LUX (by Thermo Scientific, Waltham, MA, USA), a multimode microplate reader [11]. The structural morphology and size of the different ZnO NPs and QDs were studied by Tecnai G2, FEI cryo transmission electron microscopy (TEM), operated at 120 kV (Hillsboro, OR, USA) and by dynamic light scattering (DLS) using a Zetasizer Nano ZS (Malvern Instruments Ltd., Malvern, UK). Surface modification was characterized by attenuated total reflectance Fourier transform infrared (ATR-FTIR) spectroscopy using Thermo Scientific Nicolet iS50 (Madison, WI, USA) [26]. Elemental analysis was characterized by energy-dispersive X-ray spectroscopy (EDX) detector using MIRA3 field-emission SEM microscope (TESCAN, Kohoutovice, Czech Republic) operated at acceleration voltage of 10 kV.

## 3. Results and Discussion 

### 3.1. ZnO-Nanomaterials Characterization 

**Figure 1:**
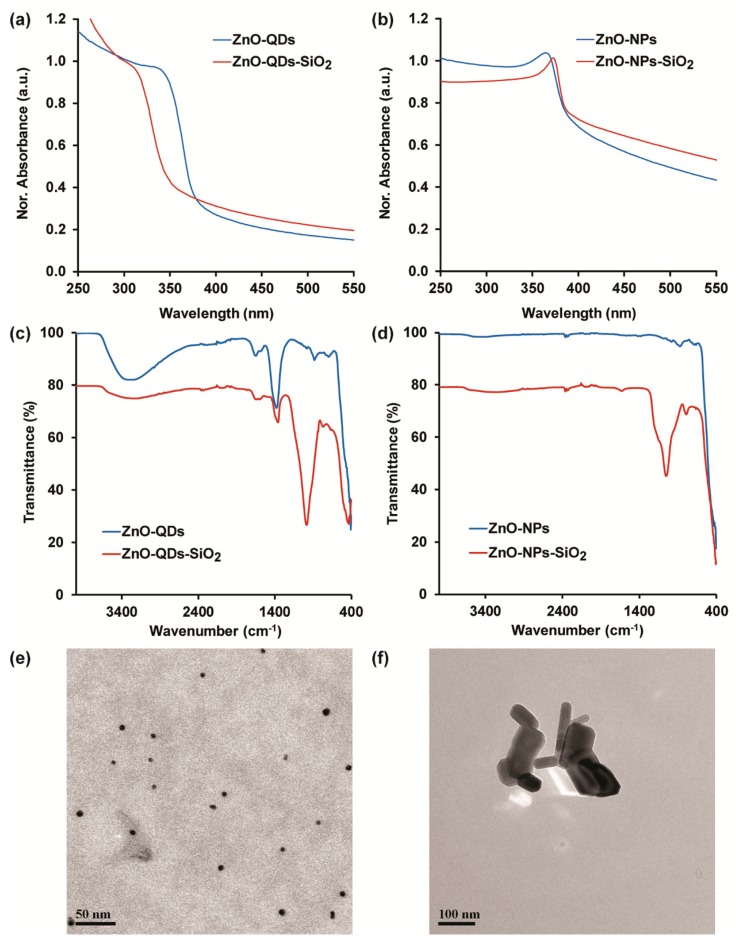
ZnO nanomaterial characterization before and after tetraethyl orthosilicate (TEOS) modification. UV-VIS spectra of (**a**) ZnO-QDs and (**b**) ZnO-NPs; attenuated total reflectance (ATR)-FTIR spectra surface characterization of (**c**) ZnO-QDs and (**d**) ZnO-NPs; Representative TEM images of (**e**) ZnO-QDs-SiO_2_ and (**f**) ZnO-NPs-SiO_2_.

Alkoxyl silanes are widely used for the surface modification of various nanomaterials and films for numerous applications [14,17,27]. Herein, TEOS molecules were applied on ZnO nanomaterials (NPs and QDs) via hydrolysis in aqueous media followed by polycondensation of alkoxyl groups in alkaline conditions, thus resulting in silica-coated ZnO-nanomaterials [18,21]. The addition of silica molecules stabilizes the colloidal aqueous solution and inhibits the time-dependent nucleation and precipitation of adjacent nanomaterials [18,20]. Surface modifications of both nanomaterials were characterized by UV-VIS spectroscopy. Figure 1a,b show the absorbance spectra of ZnO-QDs and ZnO-NPs before and after TEOS coating, respectively. The characteristic absorbance shoulder peak of ZnO-QDs blue shifts from 340 to 310 nm upon silica-coating (Figure 1a). The shift can be ascribed to the decrease in particle diameter due to the quantum confinement effect upon TEOS modification, as previously shown [16,22,28,29]. An opposite optical trend is shown for ZnO-NPs upon similar surface modification, where a red shift from 364 to 372 nm was obtained in the resulting ZnO-NPs-SiO_2_ upon mild augmentations in particle size (Figure 1b). The silica capping was further characterized by ATR-FTIR studies shown in Figure 1c,d for ZnO-QDs and ZnO-NPs, respectively. The characteristic absorption peaks of as-prepared ZnO-QDs depict a Zn-O stretching vibration at 421 cm^−1^, hydroxyl groups stretching vibration at 1649 and 3273 cm^−1^, and nitrate precursor residues peak at 1375 cm^−1^, accredited to N–O vibration (Figure 1c) [14,30]. The silica capping by TEOS molecules is shown by the additional peak at 969 cm^−1^ for the ZnO-QDs-SiO_2_ spectrum, known as an asymmetric vibration of Si–O–Si [13,16]. Figure 1d depicts similar characteristic peaks of Zn–O and O–H at 416 and 3282 cm^−1^, respectively, and the addition of siloxane peak at 1053 cm^−1^ [18]. Overall, the new characteristic peaks of both ZnO nanomaterials following TEOS modification suggest a silica shell creation. To further elucidate the latter statement, elemental composition analysis by EDX detector was performed. Figures S1 and S2 present the elemental composition spectra and the relative atomic percentage of ZnO-QDs and ZnO-NPs, respectively, before and after TEOS coating. Indeed, both Si and O elements are increased following silica capping, while the Zn values are minimized for ZnO-QDs-SiO_2_ and ZnO-NPs-SiO_2_. These results are in agreement with ATR-FTIR and UV-VIS spectral analysis, verifying the silica-coating formation. Finally, the structural dimensions of ZnO-QDs-SiO_2_ and ZnO-NPs-SiO_2_ were characterized by TEM. Figure 1e depicts the spherical shaped particles within the range of 7.3 ± 0.7 nm (applicative for the quantum confinement effect range), while the silica-coated ZnO-NPs present a mixture of different sizes and shapes at approximately 84 ± 37 nm (Figure 1f). To further strengthen the physical dimensions and size distribution, DLS measurements were performed on TEOS-coated nanomaterials. Indeed, similar features are obtained for ZnO-QDs-SiO_2_ (5.2 ± 2.4 nm, PDI of 0.121) and ZnO-NPs-SiO_2_ (109 ± 33 nm, PDI of 0.405). It should be mentioned that the TEOS coating was not observed in both ZnO nanomaterials, indicating a thin surface coverage [13,16]. 

### 3.2. FL Amplification Characterization

**Figure 2:**
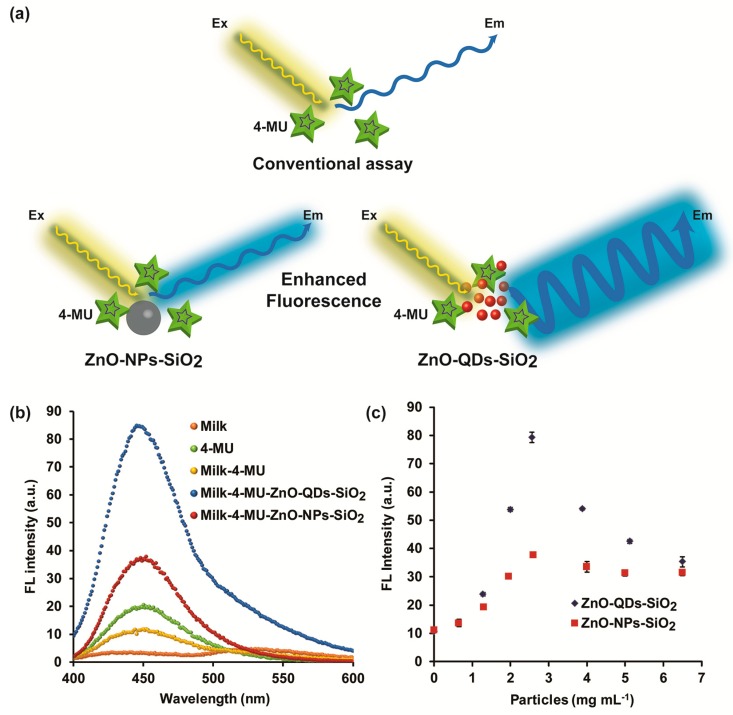
(**a**) Schematic illustration of FL signal amplification by ZnO nanomaterials; (**b**) FL emission spectra of NAGase biochemical reaction products (4-MU fixed at 20 µM) with and without ZnO-QDs-SiO_2_ and ZnO-NPs-SiO_2_; (**c**) Silica-coated ZnO nanomaterials’ content optimization of NAGase activity assay within mastitic milk. Data are reported as mean ± S.D. (n ≥ 3).

FL signal enhancement of NAGase biochemical reaction product (4-MU) was characterized in the presence of ZnO-QDs-SiO_2_ and ZnO-NPs-SiO_2_. Previous studies depict significant non-radiative energy transfer between ZnO nanomaterials to the fluorogenic reaction products [15,18]. Herein, optimized silica coverage was utilized for increased electric field modulation, considering excitation spectra overlapping and the distance-related proximity of plasmonic nanoparticles with the produced fluorophores [13,19]. Figure S3 shows the emission spectra of different TEOS amounts (0, 15, 30, 45, 63, 75 and 100 µL) used to coat ZnO-NPs using an excitation wavelength of 365 nm. The maximal FL emission for each condition is obtained at 520 nm, while the FL response is augmented with increasing TEOS coverage up 63 µL and decreased thereafter. The maximal signal (63 µL TEOS) was chosen for consecutive studies expecting pronounced FL signal amplification. It should be noted that insignificant FL values (<5 a.u.) are attained for all ZnO-NPs-SiO_2_ conditions at a characteristic E_m_ of 450 nm for the noted biochemical assay. Figure 2b presents the FL emission spectra of NAGase biochemical reaction products (4-MU fixed at 20 µM) with and without ZnO-QDs-SiO_2_ and ZnO-NPs-SiO_2_ in milk and buffer solutions, presenting maximal emission at 450 nm. Silica capping of both ZnO nanomaterials presents profound signal enhancement in milk media, above 7- and 3-fold for ZnO-QDs-SiO_2_ and ZnO-NPs-SiO_2_, respectively, in comparison to a conventional assay content omitting the addition of these nanomaterials. The insignificant FL intensity values are attained for the milk sample used as a negative control for the assay, while the positive condition (stock 4-MU) showed sufficient indicative fluorescence. The latter values were quenched upon the addition of complex media as milk due to molecular interferences [31]. Subsequently, to further optimize the bioassay efficiency in terms of signal enhancement, both optically active nanomaterials’ optimal concentrations within each microplate well assay were evaluated. Each stock ZnO nanomaterial was serially diluted in aqueous solution and added to the conventional NAGase assay for FL signal assessment [13]. The conventional assay is considered as a standard technique to evaluate NAGase activity within complex samples (i.e., milk, urine) and therefore specifically indicates udder health state [1,6,32]. Briefly, a mixture of 40 µL NAGase substrate (2.25 mM, 4-MUAG), 30 µL of mastitic milk sample and 100 µL of the different TEOS-coated nanomaterials concentrations were allowed to react, while the FL emission of the reaction products was evaluated. It is expected that an increased electric field effect is proportional to surface plasmons’ augmentation up to an optimal nanomaterial concentration for sufficient FL signal enhancement, above which the quenching effect is favored (Figure 2a) [15,19]. The latter is ascribed to intrinsic short-range molecular interactions between the produced fluorophore and the ZnO nanostructures [33,34]. Figure 2c depicts NAGase-specific catalytic reaction output within mastitic milk samples with respect to catalyst content augmentation for ZnO-QDs-SiO_2_ and ZnO-NPs-SiO_2_. Indeed, both nanomaterials’ content growth increases the FL intensities up to 2.56 mg mL^−1^, while further metal increase results in the FL signal’s gradual decline up to a maximal ZnO content. Thus, based on the pronounced signal augmentation, the silica-coated ZnO nanomaterials were set to 2.56 mg mL^−1^ and were adopted for acute phase response evaluation within different milk samples. 

### 3.3. Comparative Studies of NAGase Activity with and without ZnO-Nanomaterials 

**Figure 3:**
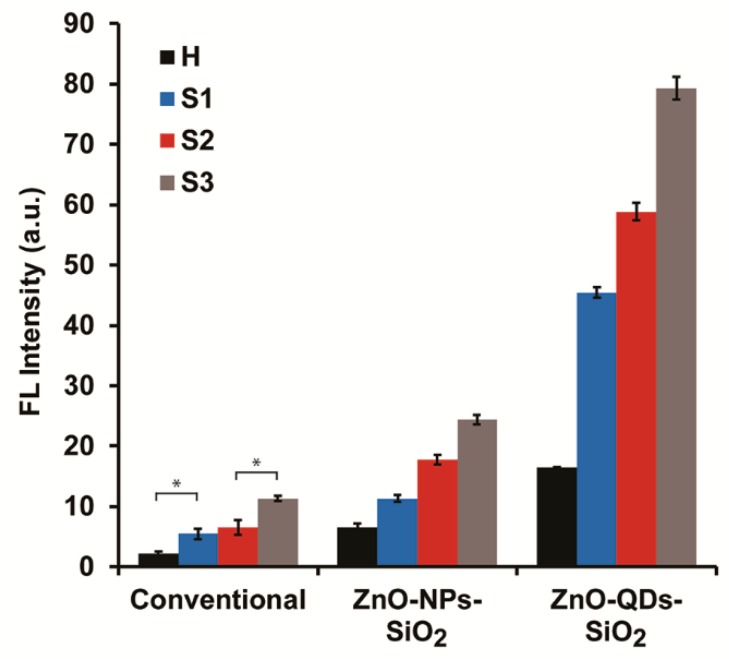
NAGase activity assay comparison in different milk qualities (healthy, subclinical and clinical BM) with and without silica-coated ZnO nanomaterials’ addition to the reaction solution. Data are reported as mean ± S.D. (n ≥ 3). * Significantly different (*t*-test, *p* < 0.05).

The developed signal amplification concept for lysosomal activity evaluations by optimized TEOS coverage and the concentration of ZnO nanomaterials in correlation to BM severity was studied in real conditions. Milk samples obtained from the Volcani Center research herd were acquired from different animals (different udders), which represent the entire inflammatory spectrum, see Table 1. *Strep. dysgalactiae*, the predominant BM causing pathogen at three SCC levels (approximately 300, 800 and >1000 (×10^3^) cells mL^−1^ identified as subclinical and clinical mastitis, respectively) were evaluated by the modified sensing concept. A healthy milk sample (H) was considered as a control condition, negative bacteriological findings and SCC lower than 100 (×10^3^) cells mL^−1^, expecting insignificant lysosomal activity by the intrinsic NAGase load. All milk samples were subjected to similar treatment conditions and were evaluated in the presence of optimized ZnO nanomaterials’ content. Figure 3 depicts the FL intensities output of NAGase catalytic activity in different milk qualities (healthy, subclinical and clinical statuses) with and without ZnO-QDs-SiO_2_’s and ZnO-NPs-SiO_2_’s independent addition to the reaction solution. Significant increases in the FL intensities are attained for augmented SCC levels in the presence of ZnO-NPs-SiO_2_ (11.3 ± 0.6, 17.7 ± 0.7, 24.4 ± 0.8 a.u. for S1, S2, S3, respectively), while sample H showed low emitted values (6.6 ± 0.6 a.u.). The increased optical response indicates the overall NAGase concentration in the analyzed samples and thus implies the severity of the occurring BM. A similar optical response is obtained for ZnO-QDs-SiO_2_ at similar escalating inflammatory statuses (45.5 ± 0.8, 58.8 ± 1.4, 79.3 ± 1.8 a.u. for S1, S2, S3, respectively), with respect to the healthy condition of sample H (16.4 ± 0.1 a.u.). These results further strengthen the correlation of NAGase lysosomal activity (FL values) as a BM-predicting indicator of various udder health conditions [2,13]. At this point, the conventional modified enzymatic assay without any addition of ZnO-nanomaterials to the reaction solution was examined. A comparable FL intensities trend is shown in correlation to SCC increase (2.2 ± 0.4, 5.5 ± 0.9, 6.5 ± 1.2, 11.3 ± 0.4 a.u. for H, S1, S2, S3, respectively) presenting escalading pathogenesis. It should be highlighted that the non-significant differentiation between S1 and S2 (*t*-test, *p* > 0.05) presents the actual limitation of the conventional assay in early-stage BM detection and severity classification. This technical drawback can be alleviated by improving the quantum efficiency and thus improving the sensitivity threshold. Overall, the optical results correspond with our previous reports and others, linking acute phase response due to an animal’s inflammatory state with augmented NAGase activity [1,6,10,35]. Both signal-amplified assays can efficiently differentiate between the various milk qualities in correlation to SCC values based on the non-radiative energy transfer reaction from the nanomaterials (ZnO-QDs-SiO_2_ and ZnO-NPs-SiO_2_) to the lysosomal reaction products (4-MU). 

Next, calibration curves for all the noted assays for NAGase activity quantification under optimized conditions for each ZnO nanomaterial were prepared using a wide dynamic range of lysosomal reaction products. Figure S4 presents the optical response of the emitted radiation in response to 4-MU content escalation (0–100 µM spiked in control milk samples). The fitted regression equations for ZnO-QDs-SiO_2_, ZnO-NPs-SiO_2_ and the conventional assay within the linear range of 0–20 µM were obtained for all assays (Figure S4). The detection limits were assessed using 3 Sa/m, where Sa is the standard of deviation and m is the slope of the linear portion, presenting values of 0.06, 0.14 and 0.32 µM for ZnO-QDs-SiO_2_, ZnO-NPs-SiO_2_ and the conventional assay, respectively. Moreover, all assays were spiked with 15 µM of 4-MU in control milk samples while evaluating the recovery efficiencies. Table S1 summarizes the recoveries of all assays with respect to the spiked concentration presenting an acceptable range of 103%–109%. Finally, the calculated enzymatic activities in the studied milk samples based on FL results are shown in Table 2. The estimated NAGase activity values support the FL studies in which lysosomal activity is increased with respect to SCC content and the severity of the induced inflammation. Significant differentiation between the inflammatory states is shown for all ZnO nanomaterials assays. NAGase activity values in the presence of ZnO-QDs-SiO_2_ overestimate ZnO-NPs-SiO_2_ for equivalent SCC levels (0.94 ± 0.02, 1.21 ± 0.03, 1.64 ± 0.04 µM min^−1^ and 0.68 ± 0.04, 1.11 ± 0.05, 1.56 ± 0.05 µM min^−1^ for S1, S2, S3, respectively). As previously mentioned, this can be accredited to the electric field effect of surface plasmons favoring quantum nanostructure over an order of magnitude in size of ZnO-NPs-SiO_2_, thus presenting higher sensitivity [15,19]. Previous studies have shown that the influence of the curvature and coupling effects of nanomaterials provides a significant near-field enhancement in which the coupling becomes more significant with decreasing particle size [36,37,38]. The conventional assay (without ZnO nanomaterials) depicts a similar activity trend for SCC escalation without the possibility of differentiating the neighboring BM statuses of S1 and S2 samples (*t*-test, *p* > 0.05). This means that the conventional assay at the current methodological form cannot be utilized for early-stage detection of subclinical BM without improving the quantum efficiency by optically active nanomaterials. Despite the slight alteration in the absolute values by the different assays, the overall trend is of importance for assessing animals’ precise inflammatory case, and therefore prescribing personal therapy treatment [3,39]. Moreover, it should be mentioned that both amplified and the conventional FL assays cannot indicate the specific bacterial species causing the BM; therefore complementary microbiological examination should be involved [6,9]. Overall, our experimental evidence played a crucial role in extrapolating the conventional NAGase assay by optically active nanomaterials, and acquiring intensified FL signals, for subclinical detection and identification. 

## 4. Conclusions 

Numerous BM diagnostic tests are routinely used to evaluate animals’ health status based on inclusive parameters. Specifically, indicative biomarkers in milk can be utilized for early BM diagnosis and drug efficacy. In the present work, we disclose enhanced FL amplification of a conventional NAGase assay, a BM-predicting biomarker, by the addition of ZnO nanomaterials. Both ZnO-QDs-SiO_2_ and ZnO-NPs-SiO_2_ induce pronounced emission within real milk samples, thus revealing significant differentiation between various somatic cell counts for convenient early bio-diagnostics and health status evaluation. The developed optimized assay can be performed in real-life conditions (untreated milk, different SCC levels and pathogens) and within the entire inflammatory spectrum (healthy, subclinical and clinical BM). Herdsman or veterinarian can utilize augmented optical indication for personalized treatment, meaning only those cows requiring attention will get it. The methodology could be applied to study many other relevant composite systems with clinical implications by monitoring indicative biomarkers through signal amplification.

## Figures and Tables

**Table 1:** Infection status and somatic cell count (SCC) values of the analyzed milk samples.

Sample *	SCC (×10^3^) Cells mL^−1^	Bacteria
H	60	N/A
S1	350	*Strep. dysgalactiae*
S2	800	*Strep. dysgalactiae*
S3	>1000	*Strep. dysgalactiae*

* Healthy milk sample (H), mastitic milk samples (S).

**Table 2:** NAGase activity in milk samples with and without the addition of ZnO-QDs-SiO_2_ and ZnO-NPs-SiO_2_ onto the reaction assay.

Sample *^,^**	NAGase Activity Conventional Assay (µM min^−1^)	NAGase Activity with ZnO-NPs-SiO_2_ (µM min^−1^)	NAGase Activity with ZnO-QDs-SiO_2_ (µM min^−1^)
H	0.30 ± 0.05	0.35 ± 0.04	0.33 ± 0.01
S1	0.73 ± 0.12	0.68 ± 0.04	0.94 ± 0.02
S2	0.86 ± 0.16	1.11 ± 0.05	1.21 ± 0.03
S3	1.50 ± 0.06	1.56 ± 0.05	1.64 ± 0.04

* Healthy milk sample (H), mastitic milk samples (S). ** *Strep. Dysgalactiae*, S1, S2, S3, Data are reported as mean ± S.D. (n ≥ 3).

## Supplementary Materials

The following are available online at https://www.mdpi.com/2079-4991/10/3/549/s1, Figure S1: EDX elemental analysis of ZnO-QDs and ZnO-QDs-SiO_2_, Figure S2: EDX elemental analysis of ZnO-NPs and ZnO-NPs-SiO_2_, Figure S3: Emission spectra of different TEOS amounts added to coat ZnO-NPs, Figure S4: Calibration curves of NAGase enzymatic activity product with and without ZnO nanomaterials. Table S1: NAGase enzymatic activity product recovery studies within control milk samples with and without ZnO nanomaterials.
