# Supplementary material for: Amplified Fluorescence by ZnO Nanoparticles vs. Quantum Dots for Bovine Mastitis Acute Phase Response Evaluation in Milk
Source: Nanomaterials (Basel). 2020 Mar 18;10(3):549. doi: 10.3390/nano10030549 (PMC7153375; doi:10.3390/nano10030549)
Supplement: Supplementary file 1 [file nanomaterials-10-00549-s001.pdf]

Supplementary electronic information

Amplified Fluorescence by ZnO Nanoparticles *vs.* Quantum Dots for Bovine Mastitis Acute Phase Response Evaluation in Milk

Narsingh R. Nirala and Giorgi Shtenberg *

Institute of Agricultural Engineering, ARO, the Volcani Center, Bet Dagan 50250, Israel; niralamn07@gmail.com
* Correspondence: giorgi@agri.gov.il; Tel.: +972-50-7795925

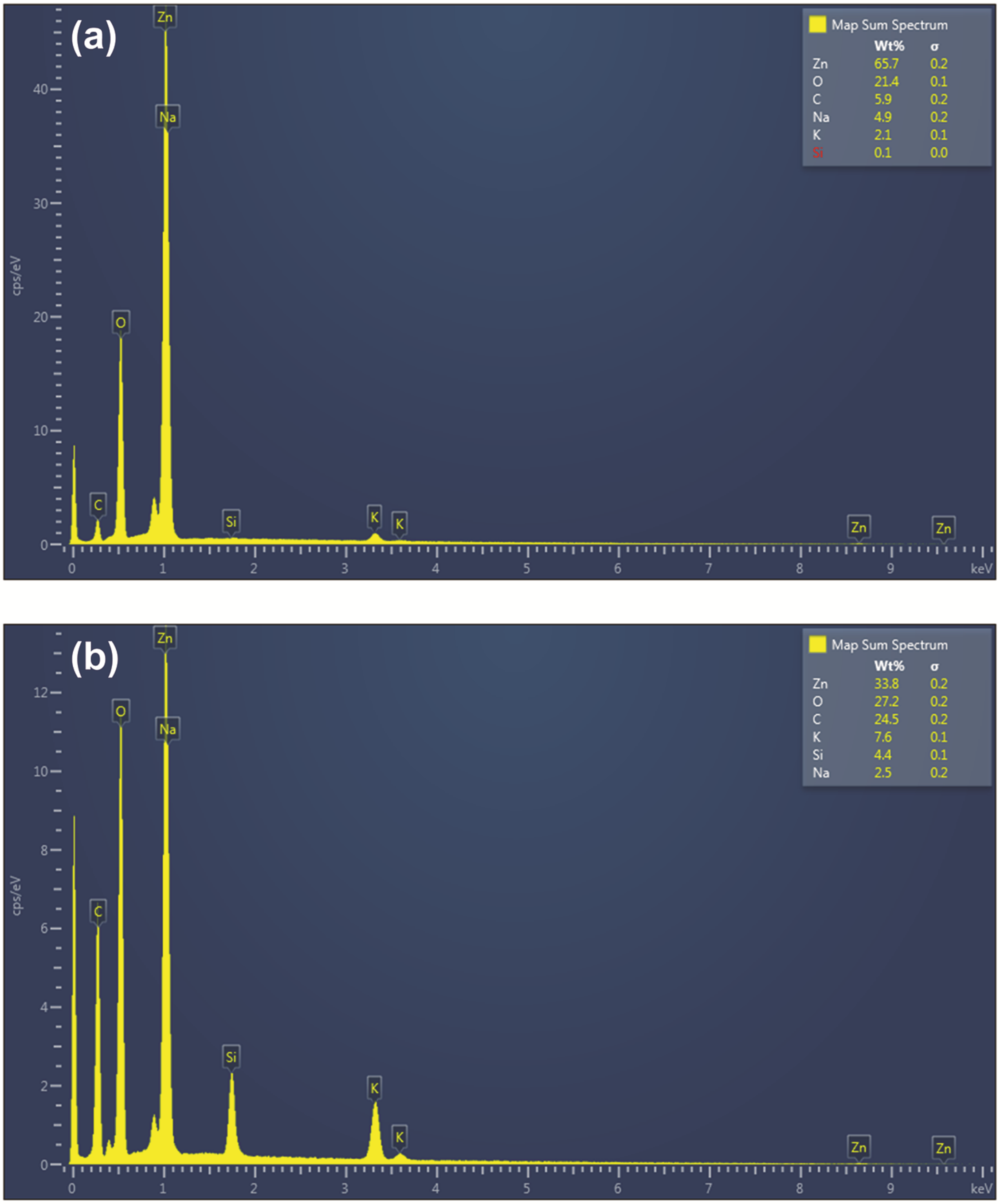

Figure S1. EDX elemental analysis of (**a**) ZnO-QDs and (**b**) ZnO-QDs-SiO_2.

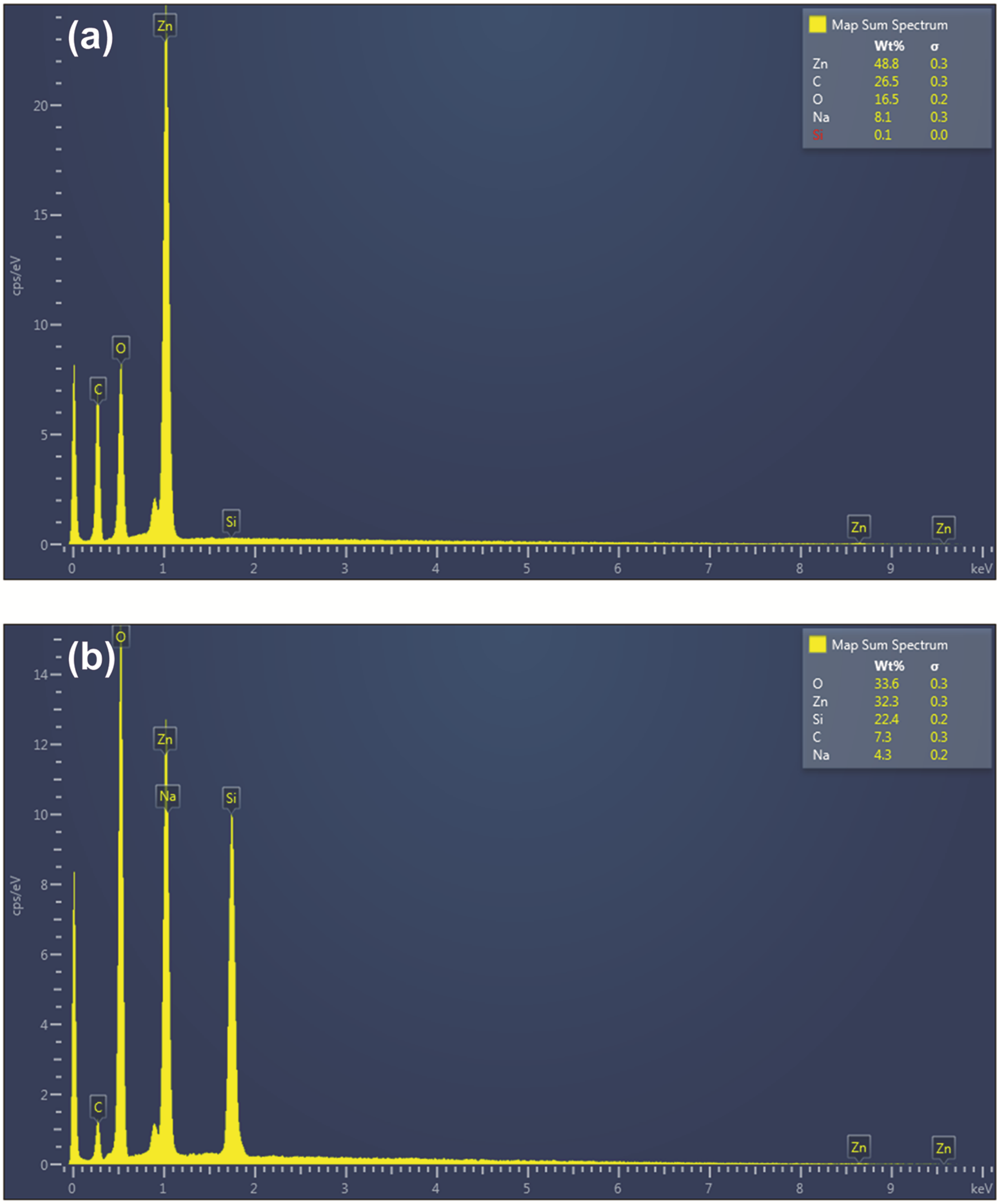

Figure S2. EDX elemental analysis of (**a**) ZnO-NPs and (**b**) ZnO-NPs-SiO_2.

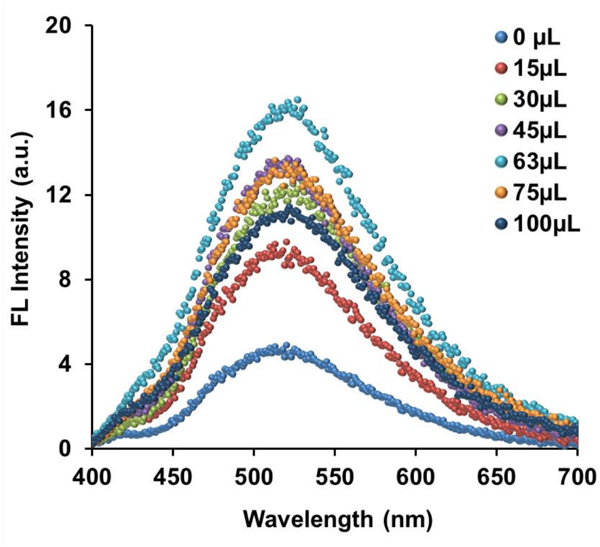

Figure S3. Emission spectra of different TEOS amounts (0, 15, 30, 45, 63, 75 and 100 μL) added to coat ZnO-NPs, according to the noted synthesis protocol of ZnO-NPs-SiO_2, under NAGase activity experimental conditions.

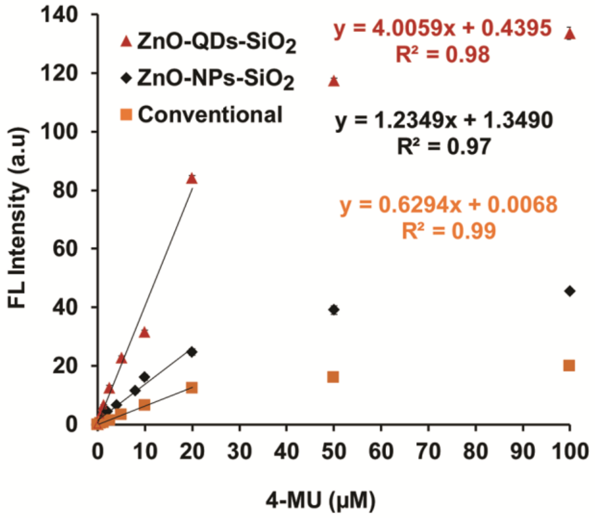

Figure S4. Calibration curves of NAGase enzymatic activity product (4-MU) emission intensities within control milk samples with and without TEOS coated ZnO nanomaterials addition to the reaction solution. Data are reported as mean ± S.D. ($n \geq 3$).

Table S1. NAGase enzymatic activity product (4-MU) recovery studies within control milk samples with and without TEOS coated ZnO nanomaterials addition.

**Assay**	**Spiked 4-MU (μM)**	**Detected 4-MU (μM)**	**Recovery (%)**
Conventional	15	15.5 ± 0.4	103
ZnO-NPs-SiO_2	15	15.7 ± 0.6	105
ZnO-QDs-SiO_2	15	16.4 ± 0.4	109
